# Successful use of therapeutic hypothermia in an opiate induced out-of-hospital cardiac arrest complicated by severe hypoglycaemia and amphetamine intoxication: a case report

**DOI:** 10.1186/1757-7241-18-4

**Published:** 2010-01-29

**Authors:** Michael Busch, Eldar Søreide

**Affiliations:** 1 Department of Anesthesia and Intensive Care Medicine, Stavanger University Hospital, Postboks 8100, 4068 Stavanger, Norway

## Abstract

The survival to discharge rate after unwitnessed, non-cardiac out-of-hospital cardiac arrest (OHCA) is dismal. We report the successful use of therapeutic hypothermia in a 26-year old woman with OHCA due to intentional poisoning with heroin, amphetamine and insulin.

The cardiac arrest was not witnessed, no bystander CPR was initiated, the time interval from the call to ambulance arrival was 9 minutes and the initial cardiac rhythm was asystole. Eight minutes of advanced cardiac life support resulted in ROSC.

Upon hospital admission, the patient's pupils were dilated. Her arterial lactate was 17 mmol/l, base excess -20, pH 6.9 and serum glucose 0.2 mmol/l. During the first 24 hours in the ICU, the patient developed maximally dilated pupils not reacting to light and became increasingly haemodynamically unstable, requiring both inotropic support and massive fluid resuscitation. After 1 week in the ICU, however, she made an uneventful recovery with a Cerebral Performance Category of 1 at hospital discharge and at a follow up examination at 6 months.

**Conclusion:**

According to most prognostic factors, the patient had a statistical chance for survival of less than 1%, not taking into account her severe state of hypoglyaemia. We suggest that this case exemplifies the need for more studies on the use of TH in non-coronary causes of OHCA.

## Introduction

Most primary survivors of out-of-hospital cardiac arrest (OHCA) will succumb to anoxic-ischemic brain injury during their hospital stay [1].

Among the factors known to predict a dismal prognosis are a non-cardiac cause of the OHCA, non-witnessed arrest, asystole as the initial ECG-rhythm, lack of bystander cardiopulmonary resuscitation (CPR) and time interval between distress call and arrival of the ambulance of more than 6 minutes [2]. Hypoglycaemic, anoxic-ischemic and amphetamine-caused brain injury share many pathophysiological pathways, such as oxidative stress, mitochondrial dysfunction, excitotoxicity, apoptosis, increased calcium influx, as well as increased seizure activity [3-7]. However, the role of therapeutic hypothermia (TH) in OHCA due to non-cardiac causes (e.g., asphyxia or drug overdose) is not widely studied [8].

## Case report

A 26-year old female sustained an OHCA after intentional poisoning. The cardiac arrest was unwitnessed, no bystander CPR was initiated, the interval from the call for help to the arrival of the ambulance and emergency physician was 9 minutes, the initial cardiac rhythm was asystole and the cause of the arrest was non-cardiac. After 8 minutes of standard advanced cardiac life support (including endotracheal intubation and i.v. injection of 2 mg epinephrine and 3 mg atropine, the patient developed a return of spontaneous circulation (ROSC). After the ROSC, the patient was haemodynamically stable and TH initiated with ice-packs. Her pupils were equal, dilated and not reactive to light. During transport to the hospital, the patient was sedated with 10 mg of diazepam due to irregular spontaneous respiratory efforts and received 500 ml of crystalloid intravenously.

Upon hospital admission, the patient's tympanic temperature was 31°C, and her ECG showed a sinus rhythm and nonspesific alterations of the ST-segment. Cerebral computer-tomography was normal. The chest x-ray showed opacification of the lower right pulmonary lobe; pO2, pCO2 and O2 saturation were within normal limits. Laboratory findings are depicted in Table 1. Drug screening was positive for opiates, benzodiazepine, amphetamine, methamphetamine and ecstasy. The patient was transferred to the *ICU *for standard post-resuscitation treatment with sedation, controlled ventilation, close metabolic control and TH. The body temperature was maintained in the TH target range (32-34°C) for an additional 26 hours before controlled rewarming was commenced.

**Table 1:** The patient's laboratory findings during the first 7 days after hospital admission

Lab findings (reference limits)	Admission	ICU Day 1	Day 2	Day 3	Day 4	Day 5	Day 6	Day 7
Troponin T (norm < 0,1 μg/l)	0,3	0,62	0,72	0,31	0,31	0,2		

Ejection fraction (norm > 53%)	30	-	-	45	-	-	55	

Vasopressor therapy	+	+	+					

Mechanical ventilation	+	+	+	+	+	+	+	+

WBC (3,8-10,8 × 10^3^/μl	32	16	15,5	7	7	8	6	

Platelets (150-450 × 10^3^/μl)	132	115	79	24	38	58	71	104

PTT (20-36 sec.)	65	45	38	36	30			

INR (norm 1,0)	1,2	1,4	1,5	1,3	1,1	1,1		

D-Dimer (< 0,5 mg/l)	>4	>4	>4	>4	>4	>4		

CRP < 10 mg/l	<1	7,5	84	126	78	46	35	

Creatinine (61,9-106 μmol/l)	182	118	140	160	114	94	88	

Base excess (± 2)	-20,7	NR	NR	NR	NR	NR	NR	NR

pH (7,35-7,45)	6,9	NR	NR	NR	NR	NR	NR	NR

Lactate (0,55-2,2 mmol/l)	17,1	NR	NR	NR	NR	NR	NR	NR

ASAT (10-45 U/l)	1927	1495	936	638	492			

NR = normal range

During glucose level control in the ICU a severe hypoglycaemia (0.1 mmol/l) was diagnosed and treated with 40 ml of 50% glucose initially. This was followed by continuous glucose infusion for 20 hours to titrate glucose levels ranging from ranged from 4.1-8.2 mmol/l. The cumulative amount of glucose infused was 102 g. When admission documentation was rechecked, it became apparent that a severe hypoglycaemia (0.2 mmol/l) had already been present at admission (i.e., 2.5 h before glucose treatment initiation). Shortly after admittance to the ICU, the patient developed maximally dilated pupils and became increasingly haemodynamically unstable, requiring inotropic and vasopressor therapy as well as substantial fluid resuscitation. Echocardiography verified a significant post-cardiac arrest myocardial dysfunction with an ejection fraction of 30%. The patient required 3 days of inotropic support before weaning was possible. After 8 hours of TH, the maximally dilated pupils decreased in size and became reactive to light. On day 2, pupillary size and reaction to light were normal.

During the course in the ICU, the patient required increasing doses of sedation, displayed spontaneous movements in all extremities and responded to endotracheal suctioning and positional change. After discontinuation of midazolam and fentanyl at day 7, she became restless with spontaneous eye opening. Shortly thereafter, she displayed purposeful motion to stimuli. Weaning from mechanical ventilation was delayed by aspiration pneumonia and bilateral pleural effusions, but the patient was extubated on day 7.

After transferral to the ward, her cerebral performance progressed continuously. The patient was graded as cerebral performance category (CPC) 1 [9] at hospital discharge. Six months later, a follow-up exam revealed no neurological or cardiovascular sequelae. The patient is currently in the second trimester of her first pregnancy.

The case is currently under investigation as an attempted homicide with the so-called "Judas-dose", a street term for the drug combination used in our patient (personal communication, Stavanger police department).

## Discussion

The good outcome in our patient was very surprising. Her statistical prognosis for survival was dismal [2,10]. Boyd noted that survival only occurred after acute poisoning leading to OHCA if 1) the OHCA was witnessed by EMS personnel or 2) the Emergency Dispatch Centre was called prior to the OHCA [11]. The prognostic data from these studies [2,10,11], however, are derived from patients not treated with TH.

The neuroprotective mechanisms of TH have been mostly studied in anoxic-ischemic brain injury, but temperature-dependent neurotoxic mechanisms of *hypoglycaemia*-, and *amphetamine- *induced brain damage have also been recognized [1,4,12-14]. No clear correlation between the additive effect of concurrent hypoglycaemic and ischemic-anoxic insults exists, partly due to the difference in the degree and distribution of neuronal necrosis of the two neurotoxins [6].

Whether the low body temperature at admission indicates possible hypothermia before the OHCA is unclear because no on-scene temperature reading was available. The protective effect of *pre*-cardiac arrest hypothermia in asphyxial CA has been established in the rat model [15]. In our experience, the vigorous prehospital application of TH measures (e.g, undressing, ice pack application and unwarmed iv. infusions) may lead to admission temperatures below 34°C for OHCA survivors.

Post-cardiac arrest myocardial dysfunction is a common but usually transient finding in OHCA survivors, and it cannot be used as a prognostic parameter [1]. In approximately 90% of patients after OHCA, s-troponin is elevated [16]. This may reflect ischemia due to insufficient perfusion during OHCA, mechanical or electrical injury due to chest compression and defibrillation or causal myocardial infarction. Myocardial ischemia and infarction have also been associated with acute insulin poisoning in the literature [17].

## Conclusion

Although our case does not prove that TH is neuroprotective in non-cardiac OHCA, we suggest that it supports the notion that TH might have an extended role in brain injury due to other aetiologies than cardiac caused, ischemic-anoxic OHCA. Our work also demonstrates that proposed prognostic factors from the pre-TH era may need to be re-evaluated as we gain more experience with the use of TH.

## Consent section

Written informed consent was obtained from the patient for publication of this case report. A copy of the written consent is available for review by the Editor-in-Chief of this journal.

## Competing interests

The authors declare that they have no competing interests.

## Authors' contributions

MB carried out the initial resuscitation, clinical follow-up of the patient and conceived the idea of possible publication of the case. MB and ES both participated equally in the literature research and the process of writing the manuscript. Both authors read and approved the manuscript.
